# Does nesfatin-1 influence the hypothalamic–pituitary–gonadal axis in adult males with obstructive sleep apnoea?

**DOI:** 10.1038/s41598-019-47061-3

**Published:** 2019-08-05

**Authors:** Halina Batura-Gabryel, Barbara Bromińska, Nadia Sawicka-Gutaj, Ewa Cyrańska-Chyrek, Barbara Kuźnar-Kamińska, Hanna Winiarska, Magdalena Kostrzewska, Ariadna Zybek-Kocik, Aleksandra Hernik, Elżbieta Wrotkowska, Lena Bielawska, Szczepan Cofta, Marek Ruchała

**Affiliations:** 1 Poznan University of Medical Sciences, Department of Endocrinology, Metabolism and Internal Medicine, Poznań, 60-355 Poland; 2 Poznan University of Medical Sciences, Department of Laboratory Diagnostics, Poznań, 60-569 Poland

**Keywords:** Respiratory tract diseases, Hypogonadism

## Abstract

There is growing evidence that obstructive sleep apnoea (OSA) influences the hypothalamic–pituitary–gonadal axis (HPG axis) in men. The aim of the study was to assess the association of nesfatin-1 with HPG axis disturbances in OSA. This is a prospective study with consecutive enrolment. It comprises 72 newly diagnosed OSA patients ((AHI: apnoea-hypopnea index) 18 subjects: 5 ≤ AHI < 15; 24: 15 ≤ AHI < 30; 30: AHI ≥ 30) and a control group composed of 19 patients (AHI < 5). All patients underwent polysomnography and fasting blood collection for nesfatin-1, testosterone, luteinising hormone (LH), high-sensitivity C-reactive protein (hsCRP), aspartate transaminase (AST), alanine aminotransferase (ALT), creatinine and glucose. Groups had similar levels of LH, nesfatin-1 and testosterone (p = 0.87; p = 0.24; p = 0.08). Nesfatin-1 was not correlated to LH (p = 0.71), testosterone (p = 0.38), AHI (p = 0.34) or the oxygen desaturation index (ODI) (p = 0.69) either in the whole group, or in sub-groups. The study did not reveal any association between the HPG axis and nesfatin-1 in OSA adult males. It is possible that nesfatin-1 is not a mediator of HPG axis disturbances in adult patients with OSA.

## Introduction

**Figure 1:**
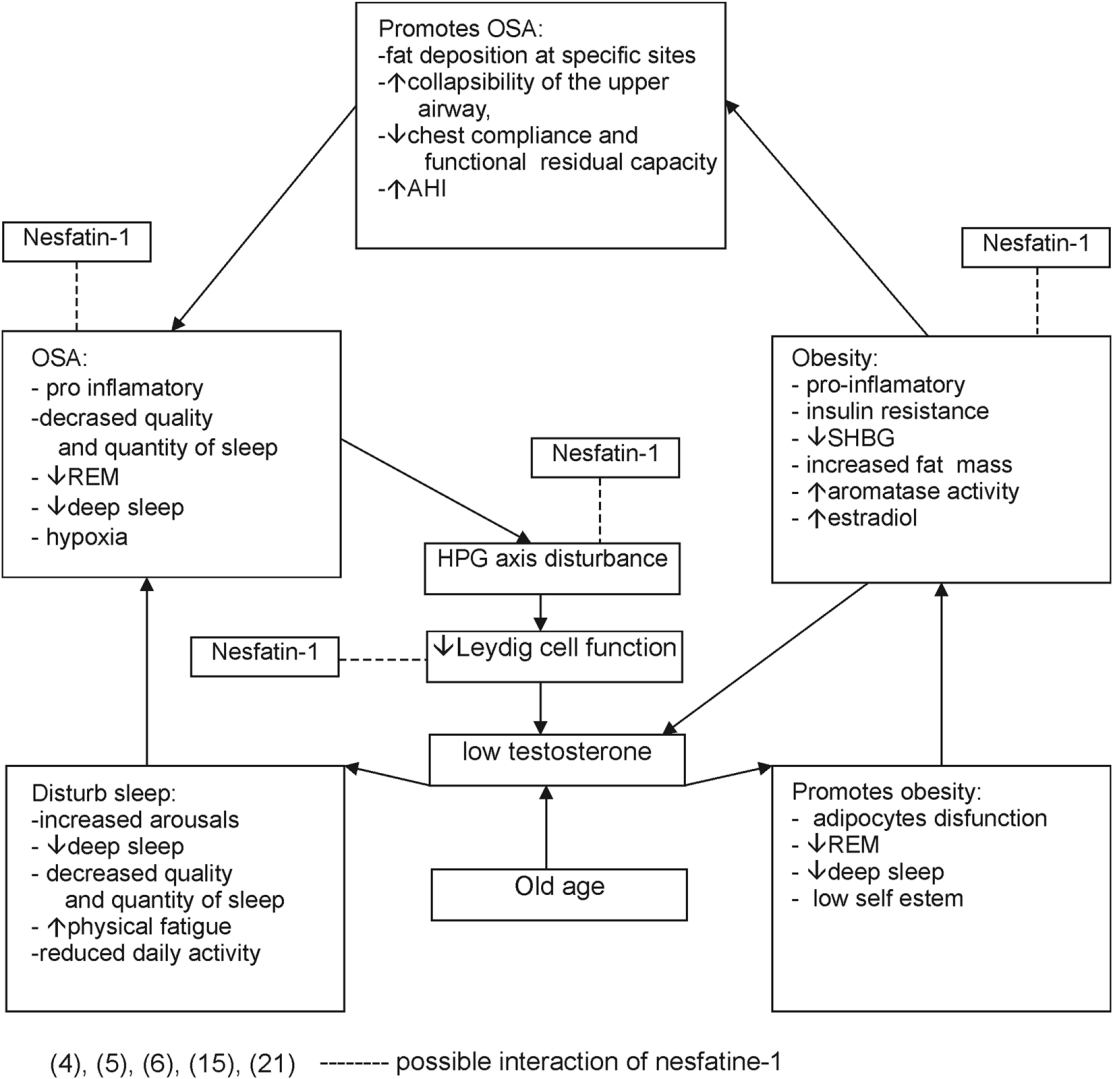
Potential mechanisms linking OSA, obesity, low testosterone and nesfatin-1. REM: rapid eye movement, SHBG: sex hormone binding globulin.

OSA is characterised by repetitive collapsing of the upper respiratory tract during sleep. It is present in about 4% of middle-aged men and in 2% of women^1^. However, this incidence is rising due to ageing of the population and increasing rates of obesity. Untreated OSA has long-term negative health consequences such as: impaired cognitive function, cardiovascular complications, insulin resistance or depression^2^. There is growing evidence that OSA influences the hypothalamic–pituitary–gonadal axis (HPG axis) and leads to sexual dysfunction in men. Testosterone is secreted in a circadian manner^3^. It is possible that OSA disturbs this rhythm via the decreased quality and quantity of sleep. A negative correlation has been noticed between OSA severity and testosterone levels^4^. Also, obesity is very common in OSA patients. Thus, low testosterone levels may be attributed to a high body mass index (BMI)^5^. Nesfatin-1 is an anorexigenic peptide created from nucleobinding-2 peptide (NUCB2), which is the *Nucb2* gene product. It exerts pleiotropic activity on the cardiovascular and digestive systems. Nesfatin-1 is involved in the regulation of stress response and behaviour. Recent studies have suggested a possible influence on reproduction. A potential feedback mechanism between NUCB2/Nesfatin-1 and the HPG axis, particularly in juvenile animal models, has been demonstrated^6^. Studies considering the influence of nesfatin-1 on the HPG axis in adult animals have provided conflicting results. Administration of nesfatin-1 either inhibits or enhances the HPG axis. There is no known potential downstream mediator of nesfatin-1. Peripherally, NUCB2/nesfatin-1 immunoreactivity has been demonstrated in human testes’ Leydig cells^6–8^. NUCB2/nesfatin-1 and melanin-concentrating hormone proteins are co-expressed in the area of the central nervous system involved in the regulation of rapid eye movement (REM) sleep^9^. A few studies have concentrated on investigating functional connections between NUCB2/nesfatin-1 and sleep. It has been found that nesfatin-1 may influence the REM phase^10^. While nesfatin-1 probably exerts an anti-inflammatory effect, OSA may interact with obesity to induce inflammation and metabolic disturbances^11^. In a study by Shen *et al*., NUCB2/nesfatin-1 was negatively correlated with the severity of OSA, BMI and waist-hip ratio^12^. Nesfatin-1 is an anorexigenic peptide, which regulates homeostatic feeding. Alotibin *et al*. revealed that nesfatin-1 levels are lower in patients with metabolic syndrome. Additionally, nesfatin-1 correlated inversely with weight, waist circumference, and BMI^13^. There is a suggestion that nesfatin-1 may even serve as an anti-obesity treatment^14,15^. The complex potential mechanisms linking OSA, obesity, hypogonadism and nesfatin-1 are depicted in Fig. 1. To sum up, low testosterone levels may disturb sleep and promote OSA. Obesity contributes to the development of OSA and disrupts testosterone production. Low testosterone levels facilitate weight gain. OSA, obesity and hypogonadism are involved in a sequence of reciprocal cause and effect. Additionally, it is possible that OSA and obesity lead to inflammation and decreases in testosterone levels^4,5^. Following the reported relationships, our study aimed to investigate the potential role of anti-inflammatory protein nesfatin-1 as a mediator of HPG axis disturbances in OSA. To the best of our knowledge, there have not currently been any studies concerning possible interactions between nesfatin-1 levels and the HGP axis in human males with OSA.

## Results

The biochemical and clinical characteristics of the groups are presented in Table 1.

**Table 1:** Biochemical and clinical characteristics of compared groups.

	Patients	Control group	*P*
Mean age (SD)	61.5 (6.6)	61.7 (5.9)	0.59
ALT U/l median (IQR)	28 (21–29.5)	29 (25.3–32.5)	0.8
AST U/l median (IQR)	23 (19–28)	20 (17.8–24)	0.22
Creatinine mg/dl median (IQR)	1 (0.9–1)	1 (0.9–1)	0.67
hsCRP mg/dl median (IQR)	1.8 (1–3.2)	1.5 (0.6–2.7)	0.51
AHI median (IQR)	**24** (**15.3–47.2)**	**2.9** (**1.1–3.8)**	**<0.00**
ODI median (IQR)	**23** (**12.9–47.3)**	**3** (**0.8–4.6)**	**<0.00**
Glucose mg/dL median (IQR)	**110** (**101–121)**	**99** (**99–113)**	**0.02**
LH mIU/ml median (IQR)	5 (3.4–6.8)	5.3 (3.8–6.2)	0.87
Nesfatin nmol/l median (IQR)	5 (4.6–6.4)	2.9 (4.5–12.7)	0.24
Testosterone nmol/l median (IQR)	12.3 (9.1–16.1)	13.4 (12.1–17.5)	0.08
BMI median kg/m^2^ (IQR)	**30.15** (**28.7–34.8)**	**27.5** (**24.7–30.4)**	**<0.00**

ALT: Alanine aminotransferase; AST: Aspartate transaminase; hsCRP: C-reactive protein; AHI: Apnoea-hypopnea index; ODI: oxygen desaturation index; LH: luteinising hormone; BMI: body mass index.

BMI and glucose differed significantly between groups (p = 0.00; p = 0.02).

Groups had similar levels of LH, nesfatin-1 and testosterone (p = 0.87; p = 0.24; p = 0.08) (Table 1). Nesfatin-1 was not correlated to LH (p = 0.71), testosterone (p = 0.38), apnoea-hypopnea index (AHI) (p = 0.34) or the ODI (p = 0.69) either in the whole group (Table 2), or in sub-groups. We also did not find any association between OSA and nesfatin-1, testosterone, and LH levels.

**Table 2:** Associations between nesfatin-1 and chosen parameters in whole studied group. Spearman’s rank correlation.

Variables	Nesfatin-1 concentrations in the serum	
	R	*P*
Age	0.08	0.49
BMI	0.03	0.8
AHI	0.11	0.34
ODI	0.05	0.69
hsCRP	−0.06	0.58
Glucose	0.03	0.76
Testosterone	0.1	0.38
LH	0.04	0.71

BMI: body mass index; AHI: Apnoea-hypopnea index; ODI: oxygen desaturation index; hsCRP: C-reactive protein; LH-luteinising hormone.

## Discussion

Previous publications have suggested the potential role of nesfatin-1 as a mediator of HPG axis disturbances in OSA. Therefore, in the present study we decided to examine possible associations between these two factors.

Our study included 72 OSA patients and a control group of 19 subjects and demonstrated similar levels of nesfatin-1, testosterone and LH. There was no association between nesfatin-1, testosterone, LH AHI and ODI.

A limited number of articles are available that discuss nesfatin-1 and its importance in the regulation of the HPG axis and they present contradictory data^16–20^. Nesfatin-1, an anorexigenic peptide, takes part in the regulation of feeding behaviour. It is created from nucleobinding-2 peptide (NUCB2), which is the *Nucb2* gene product. NUCB2 is located both centrally and peripherally. Moreover, nesfatin-1 can cross the blood-brain barrier, and possibly exert an action^21^. NUCB2/Nesfatin-1 has previously been shown to mediate between reproductive and nutritional status^8^. Additionally, in juvenile animal models, there is a functional and anatomical link between NUCB2/Nesfatin-1 and the hypothalamic–pituitary–gonadal (HPG) axis. Studies concerning the HPG axis in adult animals produce conflicting results. Preliminary data revealed that administration of large doses of nesfatin-1 increased serum levels of the LH and follicle-stimulating hormone (FSH) in male rats. This would suggest that the adult gonadotropic axis can also respond to the stimulatory effects of nesfatin-1, although its sensitivity is lower^17^. 17-estradiol and progesterone may regulate *Nucb2* mRNA expression in the pituitary gland^18^. Moreover, *Nucb2 mRNA* expression has been found in rat, mouse, and human testes. NUCB2/Nesfatin-1 protein has been discovered in interstitial mature Leydig cells^7^. These observations attest to a potential feedback mechanism between NUCB2/nesfatin-1 and the HPG axis^18^. Conversely, in a recently published study, administration of nesfatin-1 to rats and fish decreased the expression of GnRH *mRNA* in the hypothalamus and FSH and LH *mRNA* in the pituitary gland^19,20^. The authors concluded that nesfatin-1 may actually inhibit the HPG axis. To date and to the best of our knowledge, there have been no studies concerning nesfatin-1 and the HPG axis in male OSA patients. As we did not find any relationship between nesfatin-1 and the HPG axis in adult males, it might be suggested that either the adult gonadotropic axis cannot respond to nesfatin-1 or its sensitivity is lower in adults. As levels of nesfatin-1 did not differ within the studied group, we estimated that OSA severity (measured by AHI) did not influence nesfatin-1. AHI is not always sufficient to assess OSA severity. Considering the contribution of nocturnal hypoxia for mortality in OSA, ODI has an importance as a prognostic value in OSA patients^22,23^. Discrepancies between AHI and other parameters such as ODI are unclear^24^. Due to this issue, we recorded both AHI and ODI. We did not find a correlation between ODI and nesfatin-1. However, recently published research investigating this issue is equivocal. Aksu *et al*. support our findings. In their study, nesfatin-1 levels did not differ between groups divided according to OSA severity, but were significantly lower in patients with metabolic syndrome^25^. In contrast, Araz *et al*. found that OSA patients had lower nesfatin-1 levels compared to controls. Nesfatin-1 levels negatively correlated with OSA severity^15^.

We did not demonstrate differences in testosterone levels between groups. Previous studies concerning the influence of OSA on the HPG axis provided conflicting results. Gambineri *et al*.^26^ directly attributed the severity of OSA to decreased testosterone concentration, but in a study by Barrett-Connor *et al*.^27^ those associations were attenuated or disappeared after correction for waist size and BMI. Our research suggests that OSA does not influence testosterone levels. This might also be attributed to the homogenous structure of the groups – all the patients were rather obese and within the same age range.

The main limitation of this study is its small sample size. However, this is a pilot research, aiming at an initial assessment of possible interactions and the design of future prospective studies based on its results. The strength of this study is its homogenous study group. Moreover, to the best of our knowledge, this is the first study to evaluate interactions between LH, nesfatin-1, and testosterone in adult male subjects with OBS.

Previous studies have reported relationships between nesfatin-1 and gonadotropin production in the animal HPG axis, but our study did not reveal any association between the HPG axis and nesfatin-1 in adult males with OSA. It might be suggested that the adult gonadotropic axis cannot respond to nesfatin-1 or its sensitivity is much lower in adults than in younger subjects. The function of HPG axis did not differ between groups; neither did nesfatin-1 levels. it is possible that nesfatin-1 is not a mediator of HPG axis disturbances in OSA.

## Materials and Methods

### Study design and patient enrolment

This was a prospective study with consecutive enrolment. All male patients >50 years old diagnosed with OSA at the outpatient sleep department between January 2016 and September 2016 were enrolled. This study comprised 72 newly diagnosed OSA patients (18 subjects: 5 ≤ AHI < 15; 24: 15 ≤ AHI < 30; 30: AHI ≥ 30) and a control group composed of 19 patients (AHI < 5).

We excluded subjects who had previously been diagnosed with hypogonadism or pituitary disorders, patients with active neoplastic process, and patients with impaired liver or renal functions.

### Laboratory analysis

Fasting blood samples were collected for nesfatin-1, testosterone, LH, high-sensitivity C-reactive protein (hsCRP), aspartate transaminase (AST), alanine aminotransferase (ALT), creatinine and glucose. Nesfatin-1 serum levels were assessed with an ELISA Assay Kit from Sunred Biological Technology. Values of hsCRP (norm: <5 mg/dl), AST (norm: 10–37 U/l), ALT (norm: 10–41 U/l), creatinine (norm: 0.7–1.20 mg/dl), glucose (norm 70–99 mg/dl), LH (norm: 1.7–8.6 mIU/ml) and testosterone (norm: 9.9–27.8 nmol/l) were measured with the electrochemiluminescence method using Cobas 6000 (Roche Diagnostics).

Informed written consent was obtained from each participant. The study was approved by the local bioethical committee and performed in accordance with the latest version of the Declaration of Helsinki.

### Polysomnography

Complete overnight polysomnography attended by an experienced sleep technician was performed at the Sleep Laboratory in the Department of Pulmonology, Allergology, and Respiratory Oncology at Poznan University of Medical Sciences from 10 p.m. to 6 a.m. using EMBLA S4000 – Remlogic, Somnologica Studio 5.0; Natus 2009. Electroencephalograms (EEG), electromyograms (EMG), electrooculograms (EOG), electrocardiograms (ECG), haemoglobin oxygen saturation (finger pulsoximetry), the airflow through the nose and mouth (thermistor, nasal cannula), abdominal and thoracic movements, snoring sounds, and positions during sleep were observed and recorded. Apnoea was defined as more than 90% and hypopnea as at least 30% reduction of airflow for more than 10 s and associated with a decrease of more than 4% in oxygen saturation. The average number of apnoeas and hypopneas per hour of sleep was defined as the AHI. ODI was also recorded^28^. ODI was the average number of desaturation episodes per hour. A desaturation episode was a drop in the mean oxygen saturation ≥4% during 10 seconds of an apnoea-hypopnea event measured using polysomnography^22^.

### Statistical analysis

Statistical analysis was performed with MedCalc Statistical Software version 18.10.2 (MedCalc Software bvba, Ostend, Belgium; http://www.medcalc.org; 2018). The D’Agostino–Pearson test was used to assess normality. Data that did not follow normal distribution were compared with the Mann-Whitney test, while the independent samples t test was used when data followed normal distribution. Relationships between data were analysed with Spearman’s rank correlation. Logistic regression was used to analyse associations between OSA and biochemical parameters. *P* values less than 0.05 were considered significant. The datasets analysed during the current study are available from the corresponding author on reasonable request.

### Compliance with ethical standards

#### Ethical approval

The study was approved by the Bioethical Committee of Poznan University of Medical Sciences. All procedures performed in the study involving human participants were in accordance with the ethical standards of the institutional and national research committee and with the 1964 Helsinki declaration and its later amendments.

#### Informed consent

Informed consent was obtained from all individual participants included in the study for patient study participation and publication of identifying information and images.
